# The Strength of Vulnerability: How Does Supervisors’ Emotional Support-Seeking Promote Leadership Influence?

**DOI:** 10.3390/bs15101326

**Published:** 2025-09-27

**Authors:** Haoyu Wu, Hongjiang Lv

**Affiliations:** School of Economics and Management, Southeast University, Nanjing 211189, China; haoyu12344@163.com

**Keywords:** emotional support-seeking, leadership influence, LMX efficiency, status

## Abstract

Most academic work concludes that supervisors are typically viewed as providers rather than seekers of emotional support, as emotional support-seeking may be seen as a threat to their status in the workplace. However, this conclusion is incomplete due to two limitations in prior studies: (1) the tendency to interpret emotional support-seeking by high-status individuals as a lack of task-oriented competence, overlooking the relational dimension inherent in such behavior; and (2) neglecting subordinates’ expectations of authentic and approachable leadership prototypes. This study focuses on how and when supervisors’ emotional support-seeking enhances their leadership influence, from a leadership perspective that integrates relations-oriented and task-oriented dimensions. Drawing on a social network approach and regression analysis, we tested our hypotheses using data from 93 teams in 51 Chinese organizations, comprising 150 supervisors and 525 direct subordinates. The results reveal that supervisors’ emotional support-seeking is positively associated with their leadership influence via LMX efficiency. Furthermore, managerial competence moderates the relationship between supervisors’ emotional support-seeking and their LMX efficiency. This study challenges the prevailing assumption that emotional support-seeking undermines status, highlighting the positive impact of supervisors’ emotional support-seeking on their relationship and leadership in the workplace.

## 1. Introduction

Emotional support-seeking is an interpersonal risk-taking behavior in which individuals share distress or confusion to gain emotional understanding (Farh et al., 2010; Pluut et al., 2018). This behavior is particularly salient for high-status individuals, as it may signal vulnerability and potentially undermine subordinates’ respect (Hong et al., 2024; Gibson, 2018; Ollier-Malaterre et al., 2013). Accordingly, prior research suggests that supervisors are generally expected to provide rather than seek emotional support, especially in high power distance contexts (Meng et al., 2025; Tews et al., 2020; Berkovich & Eyal, 2018).

However, two critical issues suggest that the negative view of supervisors’ emotional support-seeking is not conclusive. Prior studies largely adopt a task-based perspective, framing such behavior as a signal of low ability to manage tasks or emotions (Zhang et al., 2024; Crane et al., 2019). This narrow focus overlooks its potential relational value: leadership influence derives not only from formal authority but also from informal relationships (Hogan & Kaiser, 2005). When supervisors share emotional needs while maintaining authority, subordinates may perceive this as a genuine attempt to strengthen ties (Collins & Miller, 1994; F. Li et al., 2014), thereby enhancing leadership influence (White et al., 2016; Graen et al., 2004).

A second reason the threat may be overstated is the lack of an integrative perspective on task- and relations-oriented leadership. While emotional support-seeking may resonate with subordinates’ expectations of authentic and supportive leadership (Jiang et al., 2022; Brooks et al., 2019), a lack of consistency with managerial competence—the ability to achieve organizational goals effectively—may undermine perceptions of authenticity. Subordinates may then question whether the behavior reflects genuine relational intent (F. Li et al., 2014) or strategic impression management (Jiang et al., 2023; Ollier-Malaterre et al., 2013; Davis et al., 2021), which in turn undermines perceptions of benevolence.

Building on these insights, our aim is to challenge overly negative perspectives on the relationship between high-status individuals’ emotional support-seeking and status effectiveness. Grounded in relations- and task-oriented leadership of implicit leadership theory (ILT), we propose that when supervisors seek emotional support from subordinates, subordinates form attributions based on their leadership prototypes and the perceived consistency with managerial competence (Lord et al., 1982; F. Li et al., 2014). Positive attributions foster alignment with supportive leadership prototypes, thereby enhancing Leader–Member Exchange (LMX) efficiency—the shortest path through which supervisors build high-quality relationships with all subordinates. Conversely, negative attributions undermine such endorsement (Gibson et al., 2018; Jiang et al., 2023), weakening both LMX efficiency and subordinates’ willingness to follow the supervisor.

We aim to make three key contributions. First, we challenge the traditional status-threat perspective of emotional support-seeking by high-status individuals. Previous studies suggest that emotional support-seeking by high-status individuals may undermine their status effectiveness (Hong et al., 2024; Gibson et al., 2018; Ollier-Malaterre et al., 2013). However, our study offers a contrasting view by emphasizing a relations-oriented leadership perspective that highlights the potential benefits of emotional support-seeking by supervisors. Second, we theoretically and empirically introduce and validate supervisors’ LMX efficiency as a key mechanism that links supervisors’ relationship-oriented behaviors to the realization of leadership influence. Unlike prior research that focuses on subordinates’ perception of LMX quality, we shift the focus to the supervisors’ LMX relationships with all subordinates, responding to the call for exploring the simultaneous influence of multiple LMX relationships within teams from the perspective of supervisors (Uhl-Bien et al., 2022; Martin et al., 2018; Graen & Uhl-Bien, 1995). Third, we provide new insights into the risks associated with supervisors’ emotional support-seeking at work. While we demonstrate the potential benefits of this relations-oriented behavior, we also caution that its implementation entails risks and requires careful consideration of the alignment between supervisors’ task-oriented and relations-oriented approaches.

## 2. Theory and Hypothesis

Leadership is not an objectively existing phenomenon but rather resides in subjective perceptions (Eden & Leviatan, 1975). Individuals typically hold a prototypical concept of leadership—an internalized belief about what leadership should look like—and evaluate real-world supervisors based on this prototype (Lord et al., 1982). Supervisors whose behaviors align with these prototypes are more likely to be perceived by subordinates as effective leaders (Epitropaki & Martin, 2004; Lord et al., 1982). Thus, implicit leadership theory explores how individuals use implicit cognitive structures to understand and evaluate leadership behaviors.

According to ILT, individuals tend to form cognitive representations or schemas of the typical traits and behaviors of leaders (Lord et al., 2001; Eden & Leviatan, 1975). These representations, often framed within an “if-then” paradigm, guide individuals’ responses when interacting with supervisors who match their leadership prototypes (Foti et al., 2017). ILT posits that evaluations of supervisors’ leadership influence are shaped not only by their actual behaviors but also by subordinates’ expectations of an “ideal leader” prototype (Lord et al., 2001). Table 1 summarizes the representative research findings on ILT.

**Figure 1:**
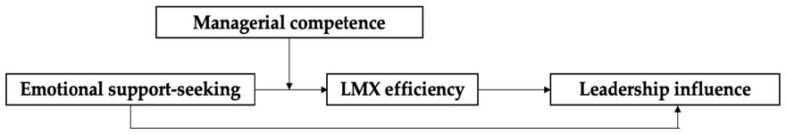
Research framework.

Building on ILT, we argue that subordinates evaluate supervisors’ behaviors through both relation-oriented and task-oriented prototypes of the “ideal leader”. The relations-oriented prototype emphasizes followers’ expectations for high-quality social exchange and emotional belonging, while the task-oriented prototype highlights followers’ expectations for leaders to drive performance and achieve goals (Heimann et al., 2020; Gerpott et al., 2019). On the one hand, supervisors’ emotional support-seeking behaviors resonate with the relations-oriented prototype, as such actions convey authenticity, approachability (Choi et al., 2022; Brooks et al., 2019), and a willingness to cultivate closer interpersonal bonds with subordinates (Collins & Miller, 1994; F. Li et al., 2014). These signals foster affective connections and relational trust, thereby strengthening leadership influence (Graen et al., 2004). On the other hand, managerial competence embodies the task-oriented prototype, reflecting subordinates’ expectations of leaders as capable, consistent, and effective in guiding work outcomes. Importantly, subordinates jointly consider both prototypes when interpreting supervisors’ emotional support-seeking (Ollier-Malaterre et al., 2013; Gibson et al., 2018). High managerial competence can validate and reinforce the positive relational signals of support-seeking, whereas low competence may undermine these signals and generate doubts about leadership effectiveness. Thus, the interplay between relations-oriented and task-oriented prototypes shapes how supervisors’ support-seeking behaviors influence subordinates’ perceptions and ultimately affect leaders’ LMX efficiency. The research framework is illustrated in Figure 1.

### 2.1. Emotional Support-Seeking and Leadership Influence

Supervisors’ emotional support-seeking can be conceptualized as a multifaceted relational behavior. From the perspective of behavioral content, it entails supervisors’ intentional self-disclosure of emotional needs toward subordinates (Farh et al., 2010). From the perspective of behavioral purpose, such acts function not merely as a means of regulating the supervisor’s negative affect (Pluut et al., 2018) but also as a mechanism for gaining emotional support and interpersonal closeness (Collins & Miller, 1994; Liu & Perrewe, 2006). From the perspective of the actor’s role, this behavior is distinctive in that it is initiated by a higher-status individual, thereby transforming hierarchical interaction into a more egalitarian exchange and signaling relational openness (Walumbwa et al., 2008).

Drawing on ILT, emotional support-seeking by supervisors offers a cognitive framework for subordinates to interpret the supervisors’ behavior, which in turn shapes their willingness to follow (Offermann et al., 1994). On one hand, when supervisors seek emotional support from subordinates, they often display emotional expressiveness (Farh et al., 2010) and a heightened concern for interpersonal relationships (Walumbwa et al., 2008). Such behaviors are likely to activate leadership prototypes characterized by warmth, humanity, and care for others (F. Li et al., 2014). Particularly, given the inherent power asymmetry that allows supervisors to conceal sensitive information, voluntary disclosure of emotional needs may strengthen subordinates’ perceptions of authenticity and humaneness (Jiang et al., 2022; Brooks et al., 2019). These prototype-consistent cues align with followers’ implicit images of trustworthy and respectable leaders, thereby enhancing their sense of belonging and attachment to the supervisor.

On the other hand, emotional support-seeking also signals supervisors’ desire to cultivate closer interpersonal bonds (Collins & Miller, 1994; Liu & Perrewe, 2006), which resonates with relations-oriented leadership prototypes. This behavior helps reduce perceived hierarchical distance and fosters psychological safety and mutual trust. Compared to merely providing emotional support, supervisors’ willingness to expose their own emotional needs may more effectively cultivate relational closeness (F. Li et al., 2014). This increased intimacy between supervisors and subordinates further reinforces the supervisors’ personal appeal and influence within the team (Graen & Uhl-Bien, 1995).

**Hypothesis** **1.** *Emotional support-seeking by a supervisor is positively related to his/her leadership influence.*

### 2.2. Supervisors’ LMX Efficiency as a Mediator

#### 2.2.1. LMX Efficiency

**Figure 2:**
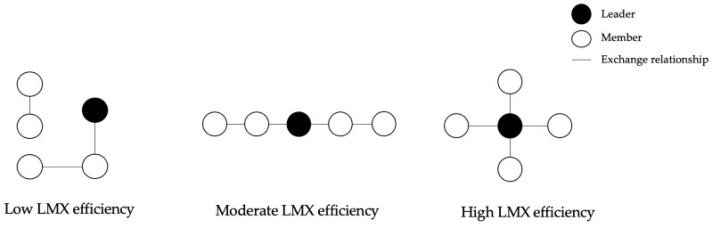
Different levels of LMX efficiency.

LMX refers to the quality of dyadic exchange relationships between supervisors and subordinates, ranging from low-quality role-based interactions to high-quality ties characterized by trust, respect, and reciprocity (Graen & Uhl-Bien, 1995; Pan & Sun, 2023). Extending beyond the dyadic view, the social network perspective conceptualizes LMX efficiency as the degree to which a leader can establish high-quality exchanges with all subordinates through the shortest paths, encompassing both direct and indirect ties (N. Li et al., 2025; Burt, 2007). Figure 2 illustrates different levels of LMX efficiency. High LMX efficiency reflects a “star-shaped” structure where the leader is directly connected to all members, signaling fairness and balance in exchange distribution. In contrast, degree centrality captures only the number of direct ties and neglects whether these ties provide efficient and equitable access to the entire team. From an ILT perspective, high LMX efficiency is more strongly associated with perceptions of effective, fair, and competent leadership than degree centrality.

#### 2.2.2. LMX Efficiency as a Mediator

When supervisors actively seek emotional support from subordinates, they project a relationally oriented leadership prototype characterized by vulnerability and approachability. First, such behaviors reduce perceptions of power distance and enhance leader approachability (Liu & Perrewe, 2006), thereby encouraging subordinates to establish and maintain higher-quality exchange relationships (F. Li et al., 2014). In traditional high power distance contexts, subordinates often perceive supervisors as authoritative, unchallengeable, and emotionally distant. By seeking understanding, comfort, or empathy from subordinates, supervisors demonstrate vulnerability and an egalitarian stance that narrows hierarchical gaps and lowers perceived power distance (Walumbwa et al., 2008). Moreover, this behavior reduces communication barriers, making supervisors appear more approachable, communicative, and trustworthy, rather than distant authority figures (Weiss et al., 2021). Second, emotional support-seeking activates reciprocity and trust mechanisms, motivating more subordinates to develop closer LMX relationships with the supervisor. Because a supervisor’s emotional self-disclosure constitutes valuable relational information, it significantly shapes subordinates’ trust in the leader (Weiss et al., 2021). Trust, in turn, is a fundamental cornerstone of interpersonal relationship building.

If a supervisor seeks emotional support only from a single subordinate, the mechanisms of approachability and trust remain confined to that dyadic relationship. However, subordinates do not automatically equate all relation-oriented behaviors with effective leadership influence (Graen et al., 2004; He et al., 2017). Rather, they evaluate such behaviors within a broader relational context, particularly considering whether the supervisor is capable of forming and sustaining relatively fair and effective relationships with all members (He et al., 2017; F. Li et al., 2014). From a team perspective, emotional support-seeking represents not merely an expression of individual vulnerability but also a bridging strategy that connects different subgroups (N. Li et al., 2025; Buengeler et al., 2021). By establishing emotional support ties with a broader range of subordinates, supervisors create additional weak ties or relational bridges across the team, which enables them to develop more direct LMX relationships with diverse members and ultimately enhances the efficiency of their overall LMX network.

**Hypothesis** **2.** *Emotional support-seeking by a supervisor is positively related to his/her LMX efficiency.*

The leadership process is shaped within the context of shared group membership, making the characteristics of the leader as a group member potentially significant in determining leadership effectiveness (Van Knippenberg & Van Knippenberg, 2005). Supervisors’ LMX efficiency can reflect their prestige and power (Luo, 2005). First, when supervisors efficiently build high-quality LMX ties with the majority of subordinates, subordinates are more frequently exposed to the supervisors’ presence, communication, and supportive behaviors. This visibility enhances the match with implicit leadership schemas, such as being communicative, supportive, and accessible (White et al., 2016). Second, perceived interactional justice strengthen supervisors’ leadership influence (F. Li et al., 2014). That is, high LMX efficiency signals relational symmetry and inclusiveness (N. Li et al., 2025), which aligns with the prototype of a fair and considerate leader. In contrast, fragmented or uneven LMX patterns may activate perceptions of favoritism, undermining the prototype-match process and thus weakening perceived influence (Hassan et al., 2013; He et al., 2017). Third, subordinates often rely on relational cues to make sense of leadership in complex and dynamic work contexts (Lord et al., 2001; Offermann et al., 1994). A structurally efficient LMX network provides consistent relational signals that guide subordinates’ interpretations of the supervisors’ intentions and capabilities, thereby reinforcing the perception of leadership legitimacy and influence (Luo, 2005; Buengeler et al., 2021).

**Hypothesis** **3.** *Emotional support-seeking by a supervisor has a positive indirect effect on his/her leadership influence via LMX efficiency.*

### 2.3. Managerial Competence as a Moderator

The implicit leadership research suggests that individuals may form their cognitive basis of a leader based on a pattern or the interaction of multiple leadership behaviors (i.e., a set of stimuli), rather than on a single behavioral trait or stimulus (Lam et al., 2015). Subordinates can comprehensively assess their leaders by activating a network of interrelated behavioral cues. When subordinates observe behaviors in which supervisors seek for emotional support, such stimuli are encoded as an initial interpretation, which generates expectations for typical leadership behavior and drives them to search for similar behavioral cues to match these expectations (Lam et al., 2015). Similarly, the managerial competence of a supervisor constitutes another critical cue for subordinates to evaluate and judge the leader prototype (DeRue et al., 2015). If the act of emotional support-seeking is inconsistent with an individual’s display of task-oriented competence, subordinates may feel uneasy and even lose trust in them (Ollier-Malaterre et al., 2013). When supervisors with strong managerial competence seek emotional support from subordinates, they may be seen as less sincere. Their behavior might be interpreted as a strategic move—either to preempt blame for potential mistakes (Davis et al., 2021) or to manage impressions and increase subordinates’ positive evaluations (Jiang et al., 2023). In contrast, when low-competence supervisors seek emotional support from subordinates, it may reinforce the cues of the supervisor’s authenticity, potentially allowing them to achieve better efficiency in supervisor-subordinate interactions, thereby increasing their leadership influence.

**Hypothesis** **4.** *Emotional support-seeking by a supervisor may has a negative indirect effect on his/her leadership influence via LMX efficiency.*

## 3. Method

### 3.1. Sample

We recruited full-time volunteers from Chinese companies in industries such as manufacturing, construction, hydropower, and electricity to enhance generalizability. With support from the human resources department, we distributed paper-and-pencil surveys to 161 supervisors and their subordinates across various functional departments, including finance, marketing, accounting, sales, and general management. The tasks were organized within traditional work groups, where members collaborated interdependently to accomplish tasks and pursue shared performance goals. All participants completed both the questionnaire A (social network) and B (organizational behavior) simultaneously, as instructed. Participants voluntarily completed the survey and returned the questionnaire in sealed envelopes to ensure the confidentiality of their responses. Researchers collected the completed surveys at the end of the day. We carefully examined each participant’s responses and excluded questionnaires with substantial missing or careless answers. When missing data exceeded 10% within a team, the entire team was removed from the dataset to ensure a response rate of at least 90%, thereby meeting the integrity requirements for whole-network data. Ethical review and approval were waived for this study in accordance with local legislation and institutional requirements.

After excluding cases with incomplete team information, final sample were 150 supervisors and 525 subordinates from 93 work teams in 51 different organizations. All teams included in this study were established at least two years prior to its commencement. Among the supervisors, 79.47% have worked in their current companies for over seven years. Regarding the subordinates, subordinates born in the 1980s account for 48.57%, while those born in the 1990s make up 32.19%, reflecting the characteristics of millennial employees. To reduce common method variance, measures such as superior-subordinate matching, random layout of questionnaires, and anonymous responses were employed during the survey. All teams had been established for at least two years prior to the commencement of this study.

### 3.2. Measures

#### 3.2.1. Social Network Measures

Emotional support-seeking, LMX efficiency and leadership influence were computed using social network methods. Specifically, social networks of emotional support-seeking, LMX and leadership influence were constructed through the participation of all team members using a social network nomination method. Participants were provided with a list of all team members along with corresponding codes and were asked to identify any members who met the criteria of the measurement items, and to provide the corresponding codes based on their own experiences. This process resulted in the creation of emotional support-seeking, social exchange relationship and leadership emergence networks for the entire team. UCINET 6.5 was used to calculate the emotional support-seeking, LMX efficiency and leadership influence for each supervisor.

Emotional Support-seeking (ESS). Using the emotional support dimension from Luo (2005), the item is: “After being criticized by your employer, from whom do you seek emotional support?”. Emotional support-seeking is measured by supervisors nominating subordinates’ IDs, with the calculation formula:

Ʃ_i_x_ij_/(n − 1), (1)

where x_ij_ is either 0 or 1, indicating whether supervisor i seeks emotional support from employee j, and n represents the team size. We calculated the extent of emotional support-seeking by the supervisor using UCINET 6.50.

LMX efficiency (LMX). We adapted the classic LMX 7-item scale from Graen and Uhl-Bien (1995) for a social network analysis approach and used closeness centrality to measure the LMX efficiency of supervisors. Closeness centrality evaluates the shortest path from one node to all other nodes in the network. If the shortest distance from a specific node (the supervisor) to all other nodes (all subordinates) is relatively small, this indicates that the node (the supervisor) occupies a central position within the LMX network. The standardized formula for LMX efficiency is as follows:

(n − 1)/Ʃdistance_ij_, (2)

where distance_ij_ represents the shortest social distance from the supervisor i to subordinate j (Luo, 2005). In practice, we used the nomination method (as described above) to construct an n*n matrix based on subordinate’ responses to the LMX-7 scale, and then used the in-closeness centrality function in UCINET 6.50 to calculate LMX efficiency.

Leadership Influence (LI). We used the social network analysis method recommended by White et al. (2016) to calculate leadership influence. The item was: “He/She is my leader, and I believe he/she plays a leadership role in my work,” and subordinates were asked to nominate supervisors they perceived as having leadership influence. The calculation formula is as follows:

Ʃ_i_y_ij_/(n − 1), (3)

where y_ij_ is either 0 or 1, indicating whether employee j perceives supervisor i as having leadership influence on him/her, and n represents the team size. We used the in-degree centrality function in UCINET 6.50 to calculate the supervisor’s leadership influence.

#### 3.2.2. Organizational Behavior Measures

Managerial Competence (MC). Supervisors reported their managerial competence by the Denison et al. (1995) five-item scale (Cronbach’s α = 0.878). Example items included “I meet managerial performance standards”, “Compared with other managers, my managerial performance is good” (1 = strongly disagree to 7 = strongly agree).

#### 3.2.3. Control Variables

Building upon prior leadership research, we selected supervisor’s gender, age, tenure, and education (edu) as control variables. Additionally, we also selected several control variables that could potentially influence the model. First, previous studies have shown that when individuals receive intimate disclosures, they tend to feel compelled to reciprocate with similar disclosures, termed the disclosure-reciprocity effect (Collins & Miller, 1994). Therefore, we included the extent to which supervisors are sought for emotional support as a control variable. The calculation formula is as follows:

Ʃ_i_Z_ij_/(n − 1), (4)

where Z_ij_ is either 0 or 1, indicating whether employee j seeks emotional support from supervisor i, and n represents the team size. We used the in-degree centrality function in UCINET 6.50 to calculate the extent to which the supervisor’s emotional support giving (ESG). Finally, considering recent research indicating that gender differences play a critical role in workplace self-disclosure and superior-subordinate relationships (Qiu et al., 2022), we controlled for the average gender composition of the team (avgen).

## 4. Results

Table 2 presents the means, standard deviations (SDs), and correlations among variables. As expected, emotional support-seeking was positively and significantly correlated to LMX efficiency (*r* = 0.221, *p* < 0.01) and leadership influence (*r* = 0.217, *p* < 0.01), and LMX efficiency was positively and significantly correlated to leadership influence (*r* = 0.446, *p* < 0.01).

The collinearity diagnostics indicated that all variables had VIF values below 5. Table 3 presents the results of regression analyses by SPSS 27. M1 reported the baseline model results. Hypothesis 1 was supported as emotional support-seeking positively related to leadership influence (*b* = 0.178, *SE* = 0.086, *p* < 0.05, M2). Hypothesis 2 was supported as emotional support-seeking positively related to LMX efficiency (*b* = 0.158, *SE* = 0.066, *p* < 0.05, M3). Further, LMX efficiency predicted leadership influence (*b* = 0.583, *SE* = 0.098, *p* < 0.001, M5). Hypothesis 3 predicted LMX efficiency mediated the relationship between emotional support-seeking and leadership influence. The findings indicate that supervisors’ emotional support-seeking enhances leadership influence through LMX efficiency (*estimate* = 0.089; *95% CI* = [0.005, 0.198]), providing further support for Hypothesis 3. Table 4 presents the indirect effects of the mediation and moderation analyses.

**Figure 3:**
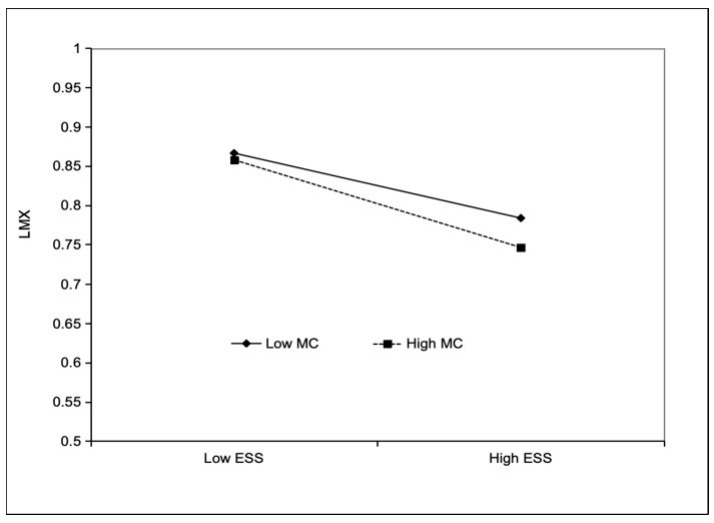
Interactive effect of emotional support-seeking and managerial competence on LMX efficiency.

Hypothesis 4 predicted that managerial competence moderated the indirect effect of supervisors’ emotional support-seeking on leadership influence via LMX efficiency. The moderating results indicated that the interaction of emotional support-seeking and managerial competence on LMX efficiency was significant (*b* = −0.036, *SE* = 0.017, *p* < 0.05, M4), with an additional variance explained of ΔR^2^ = 0.028. The results of the simple slope analysis are shown in Figure 3. The results of the moderated mediation effect analysis showed that, emotional support-seeking had no significant indirect effect on leadership influence via LMX efficiency at low levels of managerial competence (*estimate* = 0.024; *95% CI* = [−0.021, 0.074]) and no significant effect at high levels of competence (*estimate* = −0.030, *95% CI* = [−0.080, 0.008]), but the difference is significant (*estimate* = −0.054, *95% CI* = [−0.127, −0.004]). Therefore, Hypothesis 4 was partially supported. Notably, although the moderated mediation effect was small in size, it still indicates that managerial competence meaningfully shapes the direction of the indirect effect, even if its practical contribution is modest.

## 5. Discussion

Based on implicit leadership theory, we extend understanding of why and when supervisors’ emotional support-seeking promotes their leadership influence. This study shows that supervisors’ emotional support-seeking facilitates leadership influence, with LMX efficiency serving as a key mediating mechanism.

Importantly, the moderation analysis revealed a statistically significant but small effect, with conditional indirect effects at ±1 SD not reaching significance. This suggests that the moderating role of managerial competence should be interpreted as exploratory rather than conclusive. A likely explanation lies in the high power distance context of China, where managers are culturally less inclined to seek emotional support from subordinates (F. Li et al., 2014; Hong et al., 2024). The descriptive statistics for emotional support-seeking (M = 0.364, SD = 0.299) also support this observation. This cultural setting may create a “ceiling effect,” limiting the strength of the pathway from emotional support-seeking through LMX to leadership effectiveness. Nevertheless, the significant index of moderated mediation indicates that managerial competence still shapes the relative strength of this pathway (Hayes, 2015). Yet, this influence accounts for only a modest proportion of variance in leadership influence, underscoring both the cultural constraints and the exploratory nature of the finding.

### 5.1. Theoretical Contribution

Our core theoretical contribution challenges a prevailing assumption regarding the emotional support-seeking-threat for individuals in the workplace (Gibson et al., 2018; Ollier-Malaterre et al., 2013). Existing studies on social support-seeking in the workplace primarily focus on subordinates, examining the antecedents and outcomes of seeking support from supervisors (upward) or peers (lateral) (Little et al., 2017; Tews et al., 2020), with supervisors typically positioned as providers of emotional support. Addressing this gap, our findings indicate that supervisors who seek emotional support from subordinates enhance their personal influence as leaders, independent of formal positional power. By focusing narrowly on task-oriented competence, earlier work has overstated the negative implications of such behavior while overlooking its relational benefits. Our findings challenge this stereotype and extend theoretical understanding of supervisors’ proactive support-seeking in the workplace.

Second, our study advances implicit leadership theory by highlighting a relationally oriented mechanism—LMX efficiency—in supervisors’ emotional support-seeking. While prior ILT research emphasizes cognitive prototypes or task-oriented behaviors (Côté et al., 2010; J. Hu et al., 2019), we show that supervisors’ relational behaviors shape followers’ prototype activation and group-level followership. By conceptualizing leaders as integrative actors whose efficiency in managing relational networks fosters broader team influence, we bridge the gap between individual cognition and relational dynamics.

Empirically, we provide novel evidence on how supervisors’ emotional support-seeking affects team-level outcomes. Using multi-source data, we demonstrate that high LMX efficiency enables leaders to cultivate collective followership and strengthen relational networks, showing that support-seeking—often viewed as a weakness—can enhance leadership influence. This approach extends both the theoretical and methodological scope of multiple LMX research.

Finally, our study contributes to understanding the alignment between supervisors’ relation-oriented behaviors and task-oriented capabilities. Prior research has largely examined these dimensions separately, focusing on individual differences in leadership influence (Gao et al., 2024; Diddams & Chang, 2012). Our findings suggest that supervisors’ emotional support-seeking may be perceived as incongruent with high managerial competence, particularly in contexts where task-oriented capabilities are highly valued, leading subordinates to interpret such behaviors as impression management (Jiang et al., 2023). Notably, this moderation effect was small, highlighting the exploratory nature of the finding and the potential influence of cultural constraints. These results point to the need for future research to examine how alignment between multiple leadership prototypes shapes leader effectiveness.

### 5.2. Practical Contribution

First, organizations may encourage supervisors to seek emotional support from subordinates in a thoughtful manner. Doing so helps overcome the bias that vulnerability signals weakness, enhances relational leadership, and fosters a more empathetic and supportive work environment. Such practices not only improve supervisors’ psychological well-being but also strengthen their influence within teams.

Second, organizations should encourage supervisors to enhance their LMX efficiency, such as fostering high-quality exchange relationships with a broader range of subordinates. By improving leadership practices, organizations can address inequities in the distribution of LMX relationships within teams. For instance, encouraging supervisors to show increased care, guidance, and appreciation for “out-group members” can help avoid or repair unfair LMX distributions within teams, thereby mitigating existing conflicts and fostering a more inclusive and cohesive team dynamic.

Third, supervisors should carefully manage the alignment between their perceived managerial competence and their behaviors of emotional support-seeking. For lower-competence supervisors, seeking support can signal authenticity and strengthen relational ties. For higher-competence supervisors, expressing vulnerability should be performed transparently to reinforce trust and credibility. Careful calibration ensures that emotional support-seeking enhances rather than undermines leadership influence.

### 5.3. Limitations and Future Research

Our research has several limitations. First, the data were collected entirely from a Chinese context, where cultural norms regarding hierarchy and power distance may shape supervisors’ behaviors and subordinates’ interpretations. In high power distance cultures, supervisors may hesitate to display emotional needs for fear of undermining their authority, while subordinates may interpret such expressions as strategic rather than genuine. This raises questions about the generalizability of the findings to low power distance or Western contexts. Future research could therefore adopt a cross-cultural perspective to test the robustness of our results and explicitly incorporate power distance as a moderating variable. For instance, in low power distance cultures, supervisors may be more willing to reveal their emotional needs, and subordinates may perceive such behaviors as authentic expressions of humanity, thereby fostering closer supervisor–subordinate relationships. By contrast, when power distance disparities are too pronounced, supervisors’ relation-oriented behaviors may fail to elicit the expected responses from subordinates (X. Hu et al., 2024). Examining how power distance at both the individual and cultural levels moderates these dynamics would provide a more nuanced understanding of the boundary conditions of our findings.

Second, in this study, emotional support-seeking was measured using Luo’s (2005) network-based approach. However, this measurement implicitly assumes that supervisors may disclose dissatisfaction with higher-level managers to their subordinates, which could potentially undermine organizational cohesion. Considering that most measures of emotional support-seeking are derived from help-seeking (e.g., Mueller & Kamdar, 2011; Wang et al., 2023) or qualitative observations (e.g., Farh et al., 2010), they do not adequately capture the distinctive features of supervisors’ emotional support-seeking. Future research could therefore benefit from the development of more targeted measurement instruments specifically designed to assess this construct.

Third, although LMX efficiency provides a novel lens for capturing supervisors’ exchange relationships with all subordinates by incorporating both direct and indirect ties, our study relied solely on this indicator. We did not conduct robustness checks with alternative measures, such as LMX degree centrality, which primarily reflects direct ties. This reliance may limit the generalizability of our findings. Future research should systematically compare LMX efficiency with degree centrality and other network-based measures to examine whether different operationalizations of leader–member relationships yield consistent conclusions.

Fourth, due to the challenges of collecting longitudinal team network data, this study relied on cross-sectional data rather than a longitudinal design. This limitation may weaken the causal inferences that can be drawn from our findings. Future research could address this issue by employing longitudinal data or experimental designs to provide more robust evidence for the proposed causal relationships.

Last, our study focuses on the positive effects of supervisors’ emotional support-seeking. However, we did not examine whether low-status individuals could seek emotional support from high-status counterparts. Existing research has shown that individuals who receive task-related help from high-status individuals may experience heightened status threat (Tai et al., 2023). In this context, low-status individuals seeking emotional support may indeed be perceived as taking a greater risk, and such risk perceptions could hinder deeper interactions with high-status individuals. Future studies could explore the cognitive antecedents influencing low-status individuals’ willingness to seek emotional support and investigate whether this behavior might bring them certain benefits.

Furthermore, future research should expand upon our findings in several areas. First, our study specifically focuses on the behavior of emotional support-seeking, which involves sharing negative information. Prior studies suggest that individuals disclosing negative information are generally less liked than those sharing positive information; however, individuals who disclose more intimate negative information tend to be liked more than those who disclose less information (Collins & Miller, 1994). Future research could distinguish between the content and amount of self-disclosure to further explore the impact of emotional support-seeking on relationship building and leadership influence within teams.

Finally, future studies could examine mechanisms beyond relational dynamics that might explain the positive effects of supervisors’ emotional support-seeking. One key mechanism could be emotional regulation by supervisors. Disclosures of negative emotions among colleagues tend to alleviate the negative impact, while disclosures of positive emotions may enhance positive effects. However, the motivation to disclose positive emotions might be lower due to concerns that such disclosures could be perceived as boastful and thus suppressed (Hadley, 2014). Supervisors disclosing their negative emotions may help mitigate the adverse effects of such emotions. Emotional regulation could have beneficial effects on psychological well-being, emotional labor, and subsequent positive outcomes.

## 6. Conclusions

Drawing on implicit leadership theory, this study investigates how and when supervisors’ emotional support-seeking behaviors can enhance their leadership influence. Based on multi-source data collected from 150 supervisors and 525 employees across 51 Chinese companies, we find that emotional support-seeking improves supervisors’ LMX efficiency, which in turn strengthens their leadership influence. The results also suggest that managerial competence may moderate the positive effect of emotional support-seeking on LMX efficiency, such that the indirect impact on leadership influence could be weaker under certain conditions. By moving beyond the task-oriented perspective that emphasizes the potential downsides of support-seeking in the workplace, this study integrates relational and task-based leadership prototypes to extend implicit leadership theory. It also provides practical guidance for supervisors on how to seek emotional support effectively, while recognizing that the effects may be context-dependent and modest.

## Figures and Tables

**Table 1:** ILT representative research.

Theoretical Development	Representative Studies	Core Points
Introduction of ILT	Eden and Leviatan (1975)	Implicit leadership theory refers to subordinates’ beliefs about the qualities and abilities leaders should possess.
Cognitive structure elaboration	Lord et al. (1982)	Individuals typically hold prototypes of leadership; leader effectiveness may depend on the extent to which leaders meet these expectations.
	Offermann et al. (1994)	Identified eight common implicit leadership traits: sensitivity, dedication, tyranny, charisma, attractiveness, masculinity, intelligence, and strength.
	Epitropaki and Martin (2004)	Differentiated between leader prototypes (e.g., sensitivity, intelligence, dedication, dynamism) and anti-prototypes (e.g., tyranny, masculinity).
	Ling et al. (2000)	Proposed Chinese-specific leadership prototypes: moral, capability, relational, and participative leadership.
Bidirectional interaction and contextual exploration	Riggs and Porter (2017)	Emphasized cognitive interactions between leaders and followers, suggesting that leaders need to adjust behaviors to align with followers’ expectations.
	Gerpott et al. (2019); Heimann et al. (2020)	Followers hold both task-oriented and relation-oriented expectations of leaders.

**Table 2:** Means, SDs, and correlations.

Variables	Mean	SD	1	2	3	4	5	6	7	8	9
1. ESS	0.364	0.299									
2. LMX	0.805	0.224	0.221 **								
3. LI	0.635	0.301	0.217 **	0.446 **							
4. MC	5.824	0.672	0.058	0.013	−0.047						
5. Term	4.273	1.236	−0.162 *	−0.002	−0.028	0.039					
6. Gen	1.287	0.454	0.128	−0.112	−0.036	−0.001	−0.069				
7. Age	3.027	0.835	0.074	0.048	−0.079	0.291 **	0.207 *	−0.073			
8. Edu	2.987	0.768	0.054	−0.047	0.149	−0.109	−0.024	0.011	−0.156		
9. ESG	0.412	0.269	0.320 **	0.155	0.249 **	−0.016	0.036	0.068	0.040	0.094	
10. avGen	0.463	0.311	0.320 ***	0.155	0.249	−0.016	0.036	0.068	0.040	0.094	−0.194 *

Note. N = 150, * *p* < 0.05, ** *p* < 0.01, *** *p* < 0.001.

**Table 3:** Regression analysis results.

	LI		LMX		LI			
	Model 1	Model 2	Model 3	Model 4	Model 5	Model 6	Model 7	Model 8
Cons	0.506 **	0.474 **	0.847 ***	0.896 ***	−0.004	0.000	0.517	0.447 **
	(0.174)	(0.173)	(0.132)	(0.132)	(0.178)	(0.178)	(0.168)	(0.154)
Term	−0.005	0.003	0.003	0.001	−0.002	0.002	−0.001	−0.006
	(0.020)	(0.020)	(0.015)	(0.015)	(0.018)	(0.018)	(0.019)	(0.018)
Gen	−0.065	−0.078	−0.070	−0.071 +	−0.031	−0.039	−0.081	−0.044
	(0.055)	(0.054)	(0.041)	(0.041)	(0.049)	(0.050)	(0.052)	(0.048)
Age	−0.027	−0.034	0.001	0.006	−0.031	−0.035	−0.022	−0.017
	(0.030)	(0.029)	(0.023)	(0.023)	(0.027)	(0.027)	(0.030)	(0.027)
Edu	0.039	0.036	−0.019	−0.020	0.049	0.047	0.033	0.050
	(0.032)	(0.031)	(0.024)	(0.024)	(0.028)	(0.029)	(0.030)	(0.028)
ESG	0.312 ***	0.253 **	0.083	0.094	0.233 **	0.206 *	0.276 **	0.222 **
	(0.081)	(0.095)	(0.072)	(0.072)	(0.083)	(0.086)	(0.092)	(0.084)
avGen	0.146 +	0.156	−0.009	0.001	0.156 *	0.161 *	0.177 *	0.184 *
	(0.081)	(0.080)	(0.061)	(0.061)	(0.073)	(0.073)	(0.078)	(0.071)
ESS		0.178 *	0.158 *	0.047 *		0.090	0.046 +	0.018
		(0.086)	(0.066)	(0.019)		(0.079)	(0.025)	(0.023)
LMX					0.583 ***	0.561 ***		0.111 ***
					(0.098)	(0.100)		(0.022)
MC				−0.004			−0.013	−0.017
				(0.019)			(0.024)	(0.023)
ESS × MC				−0.036 *			−0.077 ***	−0.047 *
				(0.017)			(0.020)	(0.021)
LMX × MC	-							−0.037 +
								(0.020)
R^2^	0.107	0.134	0.082	0.110	0.286	0.292	0.203	0.348
△R^2^	0.107	0.027	0.082	0.028	0.179	0.006	0.069	0.145
F	2.863 *	3.129 **	1.882 +	1.931 +	8.126 ***	7.283 ***	3.969 ***	6.688 ***

Note. N = 150, + *p* < 0.1, * *p* < 0.05, ** *p* < 0.01, *** *p* < 0.001.

**Table 4:** Indirect effects of the mediation and moderation analyses.

Path		Estimate	95%CI
mediation effect (ESS-LMX-LI)		0.089	[0.005, 0.198]
moderated mediation effect	+1 SD	−0.030	[−0.080, 0.008]
	−1 SD	0.024	[−0.021, 0.074]
	difference	−0.054	[−0.127, −0.004]

## Data Availability

Due to confidentiality agreements and the sensitive nature of the data provided by some participating organizations during the survey process, we are unable to publicly share the dataset. These restrictions are in place to protect proprietary information and ensure compliance with the confidentiality terms agreed upon with the participating entities.

## Abbreviations

The following abbreviations are used in this manuscript:

LMX	Leader–Member Exchange
ILT	Implicit Leadership Theory
